# Self-consolidating concrete: Dataset on mixture design and key properties

**DOI:** 10.1016/j.dib.2024.110256

**Published:** 2024-02-28

**Authors:** Amine el Mahdi Safhi

**Affiliations:** Concordia University, Gina Cody School of Engineering and Computer Science, Montreal, Canada

**Keywords:** Experimental data, Mixture design, Self-consolidating concrete, Rheology, Workabily

## Abstract

This manuscript delineates the assembly and structure of an extensive dataset encompassing more than 2500 self-consolidating concrete (SCC) mixtures, meticulously compiled from 176 scholarly sources. The dataset has been subjected to a thorough curation process to eliminate feature redundancy, rectify transcriptional inaccuracies, and excise duplicative entries. This refinement process has culminated in a dataset primed for advanced data-driven inquiries within the SCC research domain, marking a novel contribution to the field. The dataset serves as a robust foundational resource, poised for subsequent augmentations and stringent applications in data-centric studies. It facilitates a detailed characterization of SCC properties, potentially through the implementation of machine learning algorithms, or serves as a comparative benchmark to assess the performance across diverse SCC formulations. In conclusion, the dataset serves as a crucial resource for scholars engaged in studying SCC and similar substances. It offers deep insights into the ecological benefits of substituting conventional Portland concrete with SCC alternatives. This compilation not only advances the understanding of SCC properties but also contributes to the broader conversation about sustainable construction practices.

Specifications Table

**Table:** 

Subject:	Material Characterization
Specific subject area:	Self-consolidating concrete (SCC) dataset: mixture design, material properties, performance metrics.
Data format:	Raw data
Type of data:	Table in Microsoft Excell (.xlsx and .csv format)
Data collection:	The data collection for SCC mixtures entailed a rigorous review of scholarly work, extracting key mixture designs and features. This meticulous analysis aimed to aggregate a comprehensive dataset reflecting the breadth and depth of research within this domain. The selection was predicated on a detailed consideration of the experimental methodologies employed and the material properties investigated, ensuring a broad representation of empirical data. Inclusion and exclusion criteria were strictly applied to uphold data integrity and relevance. he dataset, pending normalization, offers flexibility for future research, emphasizing empirical validity and applicability in the SCC domain.
Data source location	Concordia University, Gina Cody School of Engineering and Computer Science, Montreal, Canada. We refer to the raw data in the following link: https://zenodo.org/doi/10.5281/zenodo.10569516
Data accessibility	Repository name: A Comprehensive Self-Consolidating Concrete Dataset for Advanced Construction Practices (Zenodo) Data identification number: https://zenodo.org/doi/10.5281/zenodo.10569516 Direct URL to data: https://zenodo.org/records/10569517
Related research article	The dataset presented herein was meticulously collated through a comprehensive review of the extant literature pertaining to SCC mixtures, as delineated by Safhi et al. [1]. This process was undertaken by the current authors, who diligently curated and amalgamated the information into a unified dataset. The extraction of data was conducted from a variety of experimental outcomes delineated within the reviewed literature. These results were originally derived from an array of instruments and methodologies, alongside diverse material characterization techniques, thereby ensuring a broad spectrum of data representation. The compilation of the dataset was methodically structured, entailing the tabulation of various SCC formulations alongside their respective attributes and characteristics. This approach was aimed at facilitating the ease of analysis and interpretability. It is pertinent to note that the data acquisition strategy employed in this study was exclusively reliant on empirical and experimental data reported within the scholarly literature. There was no utilization of primary data collection methods such as questionnaires or surveys. This approach ensures the robustness and reliability of the dataset, as it is grounded in previously validated experimental findings.

## 1 Value of the Data

• *Foundation for research and development:* This dataset is an exhaustive compilation of SCC mixture designs and characteristics. It facilitates the exploration of relationships between mixture components and concrete properties, enhancing the understanding and development of new construction materials. This invaluable resource supports comparative analyses and theoretical model validations, advancing construction materials research. The dataset is a vital resource for stakeholders across construction and materials science. Academic researchers and educators in civil engineering and sustainable construction will find it invaluable for comparative studies and model validation.
• *Promotion of sustainable construction:* By including data on environmental impacts and performance metrics of SCC mixtures, this dataset plays a pivotal role in advancing sustainable construction practices. It enables the identification of greener alternatives and optimization of construction methodologies, contributing significantly to reducing the environmental footprint of the construction industry. Sustainability advocates gain a tool for promoting efficient resource use and reduced emissions in construction practices. By providing a benchmark for SCC performance and facilitating innovation, the dataset supports novel experimental protocols and the development of efficient, environmentally sustainable solutions, bridging knowledge gaps and fostering a deeper understanding of SCC applications.
• *Enhancement of machine learning (ML) applications:* The comprehensive range of data points in this dataset is ideal for training ML algorithms aimed at predicting SCC properties. This facilitates the rapid development of innovative SCC formulations with superior performance, demonstrating the dataset's potential to drive forward technological innovations in construction and materials science. Additionally, ML experts and data scientists can utilize the dataset to train models predicting SCC properties, leading to innovative formulations.
• *Benchmark for industry standards:* Offering a reliable benchmark for the performance evaluation of SCC mixtures, this dataset is an essential tool for industry professionals. It supports the objective assessment of mixture efficacies, promoting a consistent trajectory of technological advancements and quality improvement in SCC applications. Civil engineers and construction managers can leverage it to enhance project integrity and sustainability, while construction companies and developers use it to incorporate advanced, eco-friendly SCC mixtures. Policy makers and regulatory bodies will find guidance for developing standards that promote sustainable materials in construction, ensuring safety and environmental conservation.
• *Catalyst for experimental research:* The datasetʼs systematic organization and broad scope encourage the initiation of novel experimental protocols and refinement strategies. It lays the groundwork for investigating new hypotheses about SCC behavior, fostering the development of efficient and environmentally considerate concrete solutions.

## 2 Background

Self-Consolidating Concrete (SCC) is a flowable, non-segregating concrete able to spread into place, fill the formwork, and encapsulate reinforcements without the need for mechanical vibration [2,3]. Developed to address difficult casting conditions, SCC's superior flowability and stability make it ideal for complex structures and intricate reinforcements [4,5]. It offers significant benefits, including improved finish quality, faster construction, reduced labor costs, and enhanced durability, making it a preferred choice for a wide range of modern construction applications [6,7]. The deployment of data-driven approaches is increasingly crucial in unveiling the full potential of SCC and advocating for greener and more innovative construction practices.

While the latest advancements in SCC research are not encompassed by this compilation, the criteria for the selection and enhancement of the data for this publication have been rigorously considered. The importance of recent studies and contributions in the field is recognized, yet the primary focus of this compilation is on the optimization of data usefulness for the SCC research community. Meticulous curation, standardization, and presentation of a comprehensive dataset are involved. Instead of comparing our results to the latest findings, the aim of this compilation is to provide a resource that is underpinned by the reproducibility and reusability of data. It is believed that this focused approach significantly contributes to the publication's value and fosters the advancement of sustainable construction practices using SCC.

## 3 Data Description

In this paper, a compilation of SCC mixtures and corresponding properties has been utilized as the data source. Integration of the underlying data into a single table has been performed. While the primary focus of the dataset is on mixture proportioning, the inclusion of fresh properties is also featured. Other crucial characteristics of SCC, such as rheological properties (i.e., yield stress and plastic viscosity), have been investigated in several studies within the dataset. These attributes have been incorporated into the dataset where feasible, although their coverage might not be as thorough as desired (e.g., the type of aggregates). Future research that emphasizes these properties would undoubtedly contribute to a deeper understanding of the subject matter. The data can be accessed in reference [8].

The dataset offers a comprehensive summary, detailing the source references, total mix count, mixture composition, derived characteristics, fresh attributes, and rheological aspects. Encompassing more than 2500 mixtures, the dataset provides a description of each, including shared features, specific mixture formulations, as well as fresh and rheological properties. The features within the dataset are organized into seven distinct categories, each represented in the format: category name and description (number of features in the category).

1. *Identification Features* (5 features): The feature “Ref.” denotes the reference number from which the mix is sourced which can be found in the second sheet. The number of the mixture is presented alongside with the “Source” which is aligns precisely with the enumeration used by Safhi [8] and serves as a universal identifier for each SCC formulation. The year of publication was provided, and the papers were arranged by this. In cases where an internal identifier for the SCC mixture exists within the reference, it is documented under “Mixture Code” (if not, denoted by *Fn* where n is the number of the formulation).
2. *Powders Type, Content, and Density* (76 features): This section encompasses the classification and specific gravity of the utilized powders. It includes the content and density of each powder type, encompassing cement, various supplementary cementitious materials (SCMs), and other mineral additions. The SCMs included fly ash, ground granulated blast furnace slag, palm oil fuel ash, pulverized fuel ash, silica fume, pumice, natural zeolite, perlite powder, sugarcane bagasse ash, glass powder, rice husk ash, natural pozzolan, mine and quarry sludge/dust, kaolin/metakaolin, ceramic powder, masonry residue, eggshell powder, hydrated lime, nanomaterials, marble powder, dredged sediments, and limestone/dolomite powder among others. The mineral additions included fine powder (micronized calcite, fine limestone, carbonaceous/siliceous stone wastes, calcium carbonate powder, and pumice powder), limestone powder, waste ceramic powder, residue of masonry powder, and marble powder, etc. Total SCMs and total mineral addition were provided.
3. *Paste Properties* (8 features): This category is dedicated to detailing the characteristics of the concrete paste. It encapsulates the total amount of powder used, the water content, the calculated volume of the paste, the water-to-cement ratio (*w/c*), the water-to-binder ratio (*w/b*), the water-to-powder ratio (*w/p*), the volume of water to the volume of powder ratio (*Vw/Vp*), and the volume of water to the volume of cement ratio (*Vw/Vc*). Each feature provides crucial insights into the composition and consistency of the SCC paste.
4. *Aggregate Properties* (7 features): This section focuses on the characteristics of fine and coarse aggregates used in the concrete mixture. It comprises seven features detailing the content and density of these aggregates. Additionally, the total aggregate (kg/m^3^) is included for convenience, despite its redundancy, to facilitate direct access to this data without additional calculations. Furthermore, the maximum size of the aggregate (MSA) is documented alongside the fine-to-total-aggregate ratio, providing a comprehensive overview of the aggregate composition in the mix.
5. *Admixture Properties* (3 features): This category delves into the specifics of admixtures incorporated into the SCC mix. Predominantly, the admixtures accounted for across all referenced sources are superplasticizers, occasionally coupled with a viscosity modifying agent (VMA). Three features are outlined: the quantity of admixture used (in kg/m^3^), and its proportion relative to the cement and the total binder content. These features collectively provide a detailed quantitative assessment of the admixture components in the concrete formulation.
6. *Fresh Properties* (13 features): This category is dedicated to capturing the essential characteristics of the concrete in its fresh, unhardened state. It includes a total of 13 features that are divided into three key areas. The first of which covers the filling ability properties, such as the slump flow spread, V-funnel flow time, and the T_50_ time. The second area details the passing ability properties, including the J-Ring flow spread, L-box H_1_/H_2_ ratio, and U-box flow. The third area focuses on segregation resistance, featuring measurements like the sieve segregation index, column segregation index, dynamic segregation index, segregation factor, and sieve GTM stability test. Additionally, the percentage of air content is also documented. Together, these features comprehensively describe the fresh properties of the SCC, providing insights into its workability, stability, and overall performance before setting.
7. *Rheological Properties* (3 features): This section delves into the intrinsic flow characteristics of the concrete, specifically focusing on two critical features: yield stress and plastic viscosity. The third feature is the type of the used rheometer. The instruments employed in these measurements include the ICAR Rheometer, R/S Plus Rheometer, ConTec5 Viscometer, ConTec4SCC, Concrete Shear Box, and TR-CRI Concrete Rheometer. Each device offers its unique approach to assessing the rheological behavior of the SCC, contributing to a detailed understanding of its flow and deformation under stress.

In the top four lines, the minimum and maximum values were provided, as well as the average and the standard deviation. It is worthy to mention that for some papers, the data was extracted from figures. In the second sheet in the file displays the references in the following form: authors, year, title of the paper, name of the journal, volume, issue, page numbers, and the DOI.

## 4 Experimental Design, Materials and Methods

### 4.1 Validation and curation of data

The approach to validating and curating the data entailed a meticulously structured workflow, which guaranteed the development of an extensive, precise, and accessible dataset for subsequent research and analysis within the SCC domain. The methodological framework for this project, including paper selection criteria and the selection timeframe, draws from the foundational work by Xe et al. [9]. The latter database was improved, updated, and refined. This approach is further developed by refining the dataset and addressing any discrepancies, enhancing its overall applicability and significance for researchers. The SCC mixtures with no reported slump flow or rheological properties were not considered (as the self-consolidation was not confirmed).

The initial step involved removing superfluous features, like lines conveying identical information, thereby streamlining the dataset, and omitting extraneous data points. Key fields that aid in subsequent use, including total powder, paste volume, total aggregate, various ratios, and additional percentages, were retained to maintain the dataset's accessibility and informativeness. Following this, outliers were pinpointed and meticulously reviewed within the original reference source. Any potential transcription errors discovered during this process were rectified to preserve the dataset's overall precision. In instances involving the use of multiple binders, each was documented along with its specific gravity. Throughout the process, instances of mixtures documented in multiple studies were identified, and any duplicate records were eliminated to prevent repetition. This critical step ensured the dataset's coherence and maintained the distinctiveness and significance of the data.

The dataset's accuracy was improved by carefully analyzing the specific methods used to test segregation resistance and the rheometers employed for rheological property measurements. All unit measurements in the dataset were meticulously verified to ensure uniformity, making it easier for future researchers and industry professionals to use. Additionally, mixtures created from numerical interpolations were intentionally omitted to maintain the dataset's relevance to real-world scenarios and prevent skewing its overall usefulness and relevance.

Through this rigorous and ongoing process, the data validation and curation methodology has produced a dataset that is not only comprehensive and precise but also highly valuable, offering significant contributions to ongoing research and development in the field of SCC.

### 4.2 Evolution of the data

The dataset introduced in this study is not merely a static compilation of existing data, but a foundational resource intended for continuous growth and refinement. Recognizing the rapid pace of innovation in SCC research and application, we have structured the dataset to be inherently flexible, allowing for seamless integration of new data, findings, and advancements in the field. This approach ensures that the dataset remains a current and valuable resource for researchers, engineers, and industry professionals engaged in the development and application of SCC.

To facilitate the ongoing evolution of the dataset, some mechanisms were established for regular updates and community contributions. These include:

• Open access platform for data contribution: A dedicated online platform where the SCC community can submit new data and findings for inclusion in the dataset, subject to a rigorous review process to maintain data quality and relevance.
• Regular review and update cycles: An established schedule for dataset review and updates, ensuring that the latest research findings and technological advancements are reflected.
• Community engagement initiatives: Engagement with the SCC community through workshops, conferences, and online forums to gather insights, feedback, and contributions, fostering a collaborative environment for dataset enhancement.

These measures are designed to maintain the dataset as a dynamic resource that adapts to the evolving landscape of SCC research and practice. By enabling the continuous addition of new data and insights, the dataset serves as a catalyst for innovation, facilitating data-driven research and the development of novel SCC formulations and applications.

## 5 Limitations

The compilation, which spans a limited period, is not exhaustive and might inadvertently omit significant studies. Additionally, the specific types of superplasticizers and aggregates used were not considered in the dataset, which may affect the comprehensiveness of the data. The publication of this dataset is expected to prompt its review and augmentation by the research community, addressing these and other potential gaps.

### 5.1 Utilization of the data

This dataset serves as a vital component in establishing a foundational benchmark for a wide array of stakeholders in the SCC community. It primarily aids in material characterization by identifying existing research gaps and providing essential data for algorithms aimed at constructing predictive models. In the industrial realm, the dataset functions as a reliable criterion for assessing the performance of different SCC concretes, promoting continual advancement in various sectors.

The dataset ensures consistent representation of each feature by using uniform measurement units. However, to minimize potential bias in their importance in predictive modeling, future users should consider normalizing these features. The dataset adheres to the international metric system for all other measurements. Since the dataset is a compilation, not every feature is present for each formulation. Users are advised to selectively filter and tailor the dataset according to their specific needs to maintain internal consistency.

## Declaration of Competing Interest

The authors affirm that there are no existing financial conflicts or personal affiliations that could be perceived as having influenced the findings presented in this manuscript.

## Data Availability

A Comprehensive Self-Consolidating Concrete Dataset for Advanced Construction Practices (Original data) (Zenodo).
